# Response of Tahitian Bridal Veil (*Gibasis pellucida*) and Small-Leaf Spiderwort (*Tradescantia fluminensis*) to Postemergence Herbicides under Greenhouse Conditions

**DOI:** 10.3390/plants13111513

**Published:** 2024-05-30

**Authors:** Ping Yu, Stephen Christopher Marble, Patrick Minogue

**Affiliations:** 1 Department of Horticulture, University of Georgia, Griffin, GA 30223, USA; pingyu@uga.edu; 2 Department of Environmental Horticulture, Mid-Florida Research and Education Center, University of Florida, Apopka, FL 32703, USA; 3 School of Forest, Fisheries, and Geomatics Sciences, North Florida Research and Education Center, University of Florida, Quincy, FL 32351, USA; pminogue@ufl.edu

**Keywords:** weed control, invasive plant, herbicide efficacy

## Abstract

Tahitian bridal veil (*Gibasis pellucida*) and small-leaf spiderwort (*Tradescantia fluminensis*) are both invasive species in natural areas throughout Florida. However, very little is known regarding herbicide control. To provide land managers with herbicidal control options for both species, postemergence herbicides were evaluated for efficacy in a greenhouse to identify herbicide options that control both species under similar settings. Four herbicides, including triclopyr acid, triclopyr amine + 2,4-D amine, triclopyr amine, and glufosinate were applied at standard label rates and compared to a non-treated control group for efficacy. Visual control ratings were taken at 2, 4, and 8 weeks after treatment (WAT), and shoot dry weights (WAT 8) and regrowth dry weights (WAT 12) were determined. Triclopyr (acid and amine) generally provided the most consistent control of both species as evidenced by the visual control ratings and shoot dry weight data which showed reductions of 76% to 89% in shoot biomass at trial conclusion. Triclopyr + 2,4-D reduced shoot dry weights by 52% to 54% and was the least effective when considering the control of both species.

## 1. Introduction

Numerous invasive plant species are introduced in Florida and spread rapidly due to Florida’s geography, climate, and diversity in the horticultural and agricultural industries. Around 1500 non-native species have been documented in Florida, costing approximately USD 45 million annually to manage them in natural areas [1,2]. Two invasive plant species including small-leaf spiderwort (*Tradescantia fluminensis*) and Tahitian bridal veil (*Gibasis pellucida*) are prevalent in Florida and have garnered attention from land managers in recent years [3,4].

*G. pellucida* is originally found from Mexico south to South America and was introduced as an ornamental plant due to its unique growth habit and profuse flowering [5]. However, it escaped cultivation and has now been vouchered in 12 counties in Florida [2]. *G. pellucida* has also been a concern in other southern U.S. states such as Texas where it is being monitored as an invasive plant of concern [6]. *G. pellucida* is not a regulated (i.e., noxious) species nor classified as a category 1 or 2 invasive species by the Florida Invasive Species Council (FISC) but is a concern among land managers as it is likely to spread further into new areas and is difficult to manage [7]. Additionally, *G. pellucida* has been estimated to have potentially higher spreading rates compared with a similar invasive plant, *T. fluminensis* [8].

In contrast to *G. pellucida*, which could be considered a newly emerging invasive plant of concern, *T. fluminensis* has been a troublesome invasive plant in Florida for years and is currently classified as a category I invasive plant by the FISC [7], indicating that the species has been documented to alter native plant communities, change ecological functions, or hybridize with native plants. *T. fluminensis* has been vouchered in over 20 counties in Florida, primarily confined to central and north Florida [2]. Differences and similarities between the two species have been detailed previously [5,9,10]. In brief, both plants are spreading herbaceous groundcovers which root extensively along their nodes, have a high propensity for spreading via stem fragmentation, and form dense vegetative mats on the forest floor which alter the germination and growth of many understory species [4,6].

*T. fluminensis* has been the subject of numerous herbicide evaluations as it is a problematic species in Florida and other countries such as New Zealand and Brazil. Overall, triclopyr (multiple formulations) has been the most consistently effective option across many of the different studies [4,11,12,13,14], yet triclopyr can damage many native broadleaf plant species [15], leading researchers to search for other viable non-chemical alternatives. Artificial shading (reducing ambient light by 80–90%) has been shown to reduce *T. fluminensis* cover by over 60% and was less injurious to native tree seedlings compared with the use of herbicides but was not feasible in large-scale infestations [15]. Glyphosate is another potential option but has provided variable control depending upon timing, rate, and environmental conditions [11,14]. Other broadleaf active herbicides such as 2,4-D have generally been ineffective on mature populations in the field [14,16,17]. While less research has focused on managing *G. pellucida*, a recent report by Yu et al. evaluated *G. pellucida* control with nine different active ingredients including 2,4-D, 2,4-D + triclopyr amine, aminopyralid, fluroxypyr, glufosinate, glyphosate, metsulfuron-methyl, and triclopyr (applied as either acid or amine formulations) in a greenhouse setting [10]. Following eight weeks of evaluation, the data showed the most efficacious treatments consisted of all triclopyr treatments along with fluroxypyr, glufosinate, and glyphosate providing similar control. The results were similar to the previous greenhouse study, reporting *T. fluminensis* control depending upon herbicide types and rates except for glyphosate, which provided approximately 40% to 60% control [14].

**Figure 1:**
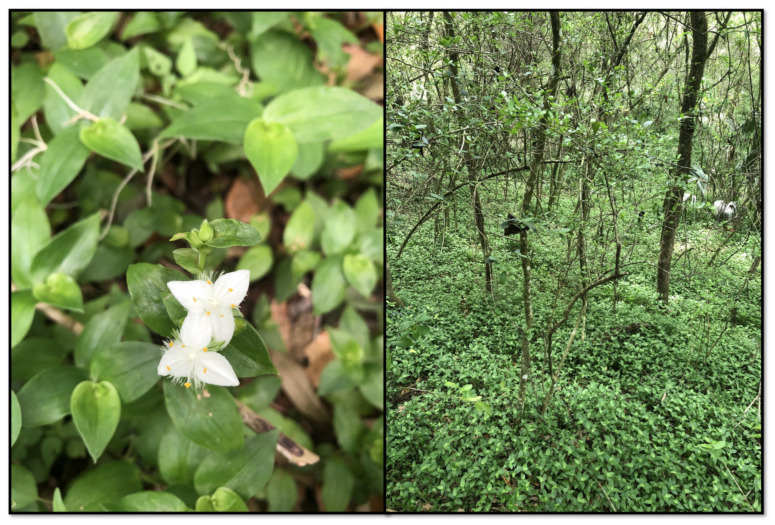
*Tradescantia fluminensis* flower (**left**) and example of spreading growth habit (**right**).

**Figure 2:**
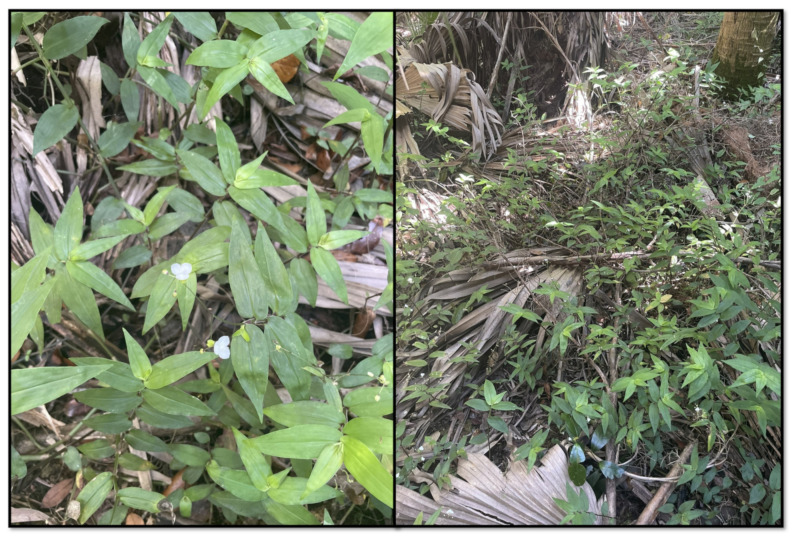
*Gibasis pellucida* in flower (**left**) and example of spreading growth habit (**right**).

Due to the growth habits of *G. pellucida* and *T. fluminensis*, it can be difficult to distinguish those two species in the field [3,5,9,10,15,16] (Figure 1 and Figure 2). As such, it would be important for land managers to be able to properly differentiate the two species and have established herbicidal control options to control both species. As no previous research has simultaneously evaluated the control of these two species, the objective of this research was to identify herbicide options that provide control for both *T. fluminensis* and *G. pellucida* under similar settings and to determine if trends in herbicidal control differed between the two species. As *T. fluminensis* has been shown to recover following herbicide treatment under both field and greenhouse settings [10,14] and the regrowth potential of *G. pellucida* has not been assessed [10], an additional novel component of this study was to assess the regrowth potential following an initial biomass assessment. The overall goal was to identify promising herbicide options that can then be evaluated in longer-term experiments on large established populations under field conditions that control both species.

## 2. Results

**Figure 3:**
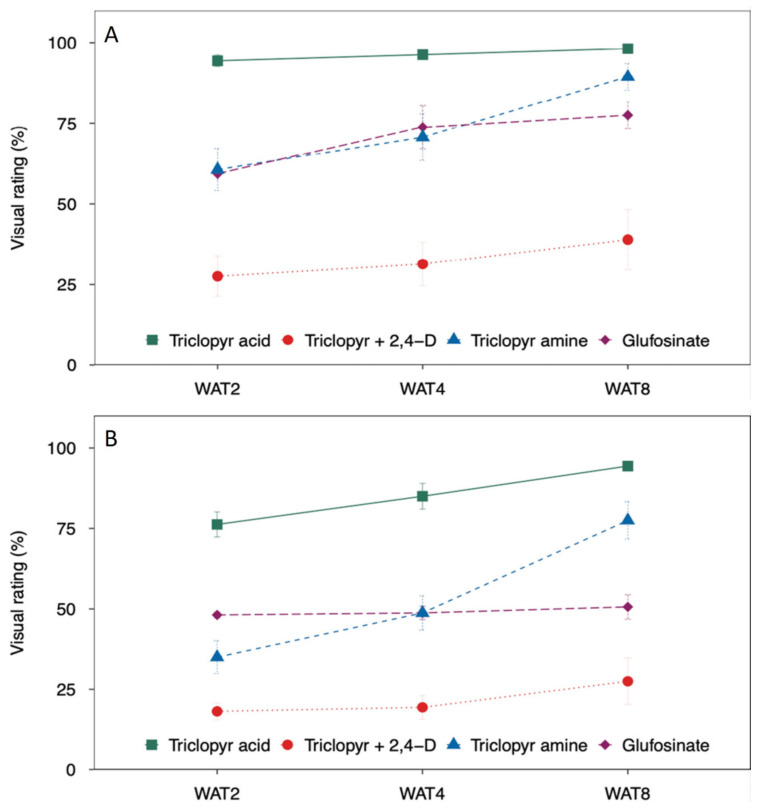
Mean visual ratings of Tahitian bridal veil (*Gibasis pellucida*) (**A**) and small-leaf spiderwort (*Tradescantia fluminensis*) (**B**) control following treatment with selected herbicides (triclopyr acid, triclopyr amine + 2,4-D amine, triclopyr amine, and glufosinate) at 2, 4, and 8 weeks after treatment (WAT). Means and standard error bars are shown and are pooled over two experimental runs.

For *G. pellucida*, triclopyr acid provided the highest visual control at the early evaluation dates 2 and 4 weeks after treatment (WAT) with over 90% control (Figure 3A). The lowest control was observed in plants treated with triclopyr amine + 2,4-D amine, reaching only 39% control by WAT 8. By WAT 8, 90% to 98% control was achieved with triclopyr amine and acid, respectively, while glufosinate provided a similar level of control (78%).

The results for *T. fluminensis* in general followed the same trend as the results observed in *G. pellucida* with the visual control ratings showing the highest control of plants treated with triclopyr acid (94% control) or triclopyr amine (78% control), followed by glufosinate (51% control), and the lowest control of plants treated with triclopyr amine + 2,4-D amine (28% control) (Figure 3B).

**Figure 4:**
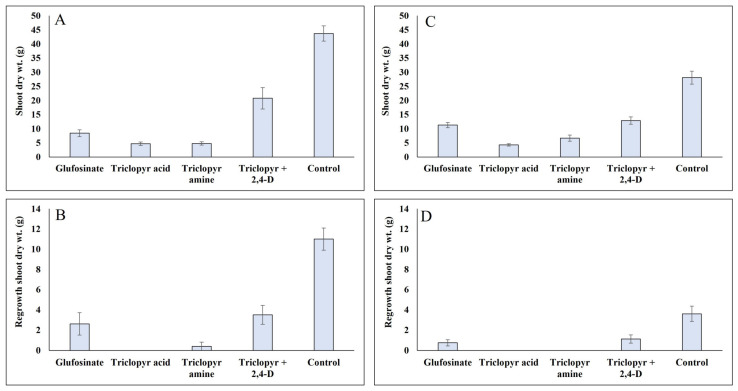
Mean Tahitian bridal veil (*Gibasis pellucida*) shoot dry weight (**A**) and regrowth (**B**) (presented in grams) following treatment with selected herbicides including triclopyr acid, triclopyr amine + 2,4-D amine, triclopyr amine, and glufosinate. Mean shoot dry weight and regrowth for small-leaf spiderwort (*Tradescantia fluminensis*) are shown in graphs (**C**,**D**). Mean shoot dry weight for both species (**A**,**C**) was collected at 8 weeks after treatment, while shoot regrowth (**B**,**D**) was collected at 4 weeks following the initial harvest (12 weeks after the initial treatment).

Shoot dry weight data showed a similar trend as visual control ratings in *G. pellucida* where triclopyr acid and amine along with glufosinate resulted in the lowest shoot dry weights Figure 4A). The results from this study are similar to Yu’s [10] where triclopyr (both acid and amine formulations) and glufosinate all provided a high level of *G. pellucida* control, outperforming other herbicides evaluated, including 2,4-D amine, aminopyralid, and metsulfuron-methyl. At four weeks following the initial shoot harvest, triclopyr acid and amine also resulted in the minimum regrowth (0 and 0.4 g, respectively), providing approximately 100% control of shoot regrowth in relation to the non-treated control group (Figure 4B).

At this time, glufosinate provided a 76% reduction in regrowth, while triclopyr amine + 2,4-D amine resulted in the highest amount of shoot regrowth but still resulted in a 68% decrease in relation to the non-treated control group. Overall, the data suggest that triclopyr (acid and amine) could provide persistent control of *G. pellucida*, significantly reducing any regrowth potential that may arise following initial treatment. Glufosinate provided a high level of visual control early in the experiment and was in general similar to triclopyr in biomass reduction. However, glufosinate caused rapid symptom development, especially when compared with triclopyr, which may have resulted in higher ratings during early evaluation periods. Additionally, as glufosinate is not fully translocated [18], further testing is warranted to determine if glufosinate performs similarly to triclopyr under field conditions using more mature populations as significant regrowth may occur.

For *T. fluminensis*, shoot dry weight data confirmed the visual control estimates with triclopyr acid and amine resulting in the lowest shoot dry weights (4.3 g and 6.7 g, respectively), equivalent to an 85% and a 76% reduction in shoot dry weight relative to the non-treated plants (shoot dry weight of 28.1 g) (Figure 4C). Treatment with either triclopyr formulation resulted in no regrowth (Figure 4D). Glufosinate resulted in a 79% reduction in regrowth (0.7 g) compared with the non-treated control (3.5 g) and was similar to both formulations of triclopyr. Triclopyr amine + 2,4-D amine-treated plants showed the greatest regrowth which was still approximately 70% less than the non-treated control.

## 3. Discussion

When comparing herbicides for *G. pellucida* and *T. fluminensis* control, the same efficacy trend was generally observed with triclopyr acid providing the best control followed by triclopyr amine and glufosinate, which performed similarly, and the lowest level of control was achieved with triclopyr amine + 2,4-D amine. Overall, the control of *G. pellucida* tended to be higher than *T. fluminensis*, based on both the visual control estimates and shoot weight measures. This suggests that the current triclopyr amine recommendation for *T. fluminensis* control would be expected to provide similar or greater control of *G. pellucida* if both species co-inhabited the same area and needed to be managed. The use of triclopyr amine would often be preferred over the use of the triclopyr ester formulation because of volatility concerns and potential damage to non-target plants. While 80 to 90% control was achieved with triclopyr in greenhouse experiments at rates as low as 1.7 kg ae triclopyr ha^−1^ [14], less than 60% control was observed in the present study using a triclopyr amine + 2,4-D amine combination with triclopyr applied at 1 kg ae ha^−1^ indicating that a higher dose is needed for consistent control. Data presented here along with previous reports suggest that close to 100% control of either species could be achieved using triclopyr acid or amine at a rate equivalent to 3.4 kg ae ha^−1^. It should also be noted that while glufosinate was found to be an effective option in this study and in previous work for both species [10,14], longer-term studies of regrowth potential are needed under field conditions. While glufosinate provided reductions similar to that of triclopyr amine or acid, glufosinate-treated plants demonstrated significant regrowth. This suggests that a control with a contact action herbicide such as glufosinate may provide less control under field conditions and possibly not be as effective as previously reported in greenhouse studies [10].

While much more research has been devoted to the herbicidal management of *T. fluminensis* under field conditions [4,14,16], there are no previous evaluations of the herbicide efficacy for the control of *G. pellucida* under field conditions. Research by Yu identified efficacious options for *G. pellucida* under greenhouse conditions. This current work is the first to report that, at least for the herbicides tested here, herbicides that have been effective for controlling *T. fluminensis* would be expected to provide similar or greater levels of control for *G. pellucida* [10]. As higher levels of efficacy are generally observed under greenhouse conditions, all findings warrant further investigation on mature populations under field conditions in order to develop effective management plans [19].

## 4. Materials and Methods

Studies were conducted in a greenhouse (60% reduction of ambient light) located at University of Florida, Mid-Florida Research and Education Center in Apopka, FL, USA in 2022. In March, terminal stem cuttings (15 to 20 cm in length) of *G. pellucida* were collected from a local park (Big Tree Park, Altamonte Springs, FL, USA, 28.7214° N, 82.3005° W), while *T. fluminensis* cuttings were collected from a state park (Payne’s Prairie Preserve, Gainesville, FL, USA, 29.6097° N, 82.3005° W). On the collection day, terminal cuttings of both species were inserted into separate sets of nursery pots (diameter at top 16.4 cm and bottom 12.5 cm, depth 17.5 cm, and volume 2.84 L) filled with a standard soil-less substrate (Southeast Soils Inc., Okahumpka, FL, USA, pine bark:Florida peat:sand = 9:1:1, *v*/*v*/*v*) with 4 stems per pot. Two weeks after sticking, 11 g of control release fertilizer (17N-2.2P-9.1K, Osmocote^®^ Blend 17-5-11, 8 to 9 months, (ICL Specialty Fertilizers, Dublin, OH, USA)) was top-dressed to each pot.

On 3 May, approximately 6 weeks after sticking cuttings (mostly cloudy skies, 26 °C, 76% relative humidity, and calm winds), each pot contained four individual fully rooted plants that were approximately 40 to 50 cm in length. At this time, all plants were removed from the greenhouse and placed onto a gravel area outdoors where selected herbicides (Table 1) were applied using a CO_2_ backpack sprayer calibrated to deliver 234 L ha^−1^ using a TeeJet 8004 flat fan nozzle (TeeJet Technologies, Wheaton, IL, USA) at 241 kpa. All herbicide treatments were selected based on previous efficacy studies conducted separately on *G. pellucida* and *T. fluminensis* and included options that would be labeled for application in and around riparian habitats [10,14]. Here, the herbicide options found to be the most effective on *G. pellucida* [10] were evaluated again to monitor longer-term regrowth and 2,4-D amine + triclopyr amine and triclopyr acid were also included to determine their efficacy on *T. fluminensis,* as they had not been tested in previous work. A non-ionic surfactant (AirCover, Winfield Solutions, St. Paul, MN, USA) was added at 0.5% (*v*/*v*) to triclopyr acid and triclopyr amine treatments based on manufacturer’s recommendations. At 24 h after herbicide treatment, all plants were moved back inside the greenhouse where they remained for the duration of the experiment. Plants were watered daily with 1.3 cm overhead irrigation (Xcel-Wobbler; Senninger Irrigation, Clermont, FL, USA) via two irrigation cycles throughout the experiment. The study was repeated following the same methodology and timeline with treatment applications made on 10 May 2022 (clear skies, 26 °C, 46% relative humidity, and calm winds).

At 2 weeks after herbicide treatment (WAT 2), plants were visually evaluated using a control rating scale 0 to 100, with 0 indicating no control (no damage) and 100 representing complete control (100% damage). Subsequent visual ratings were taken at WAT 4 and WAT 8. At the conclusion of the experiment at WAT 8, plant shoots were clipped at the soil line and shoot dry weight was determined after placing shoots in a forced-air oven at 60 °C for 7 days until a constant weight was reached. The pots were retained in the greenhouse to obtain the regrowth data. At four weeks following the first shoot harvest (WAT 12), the shoot regrowth was harvested, and the shoot dry weights were measured with the same method. The experiment was arranged in a randomized completed block design with 8 replications for each herbicide treatment, being repeated in time. In all cases, a non-treated (water check) control group of plants was maintained and used as a comparison.

Data from the two experimental runs were combined as there were no experimental runs by treatment interactions. The significance of treatment effects was determined by analysis of variance (ANOVA) using R program software version 3.5.1. Multiple comparisons were conducted using Tukey’s honestly significant difference (HSD) test at 5% probability.

## 5. Conclusions

This study confirmed previous work and showed that triclopyr provides effective and consistent control of both *G. pellucida* and *T. fluminensis* when applied at rates of at least 3.4 kg ha^−1^ in the acid or amine formulation. While previous reports showed glufosinate provided a high level of control based on visual injury ratings and shoot dry weight reductions, longer-term regrowth data taken in this study showed recovery potential and thus longer-term field studies are needed to confirm efficacy. Triclopyr + 2,4-D applied as a tank mixture significantly decreased the above-ground biomass of both species but tended to be less effective than the other options tested and would likely provide ineffective results under field conditions due to the lower triclopyr rate utilized in the mixture.

It is also important to note that in this side-by-side comparison, *G. pellucida* produced more biomass and had a higher propensity for regrowth in comparison with *T. fluminensis* when both were tested under identical conditions. Thus, while the same herbicidal options were effective for both species, land managers may need to inspect areas treated for *G. pellucida* management more frequently to ensure recovery has not taken place.

## Figures and Tables

**Table 1:** Selected herbicides evaluated for postemergence control of *Gibasis pellucida* and *Tradescantia fluminensis* in greenhouse experiments in Florida.

Herbicide	Trade Name	Rate (kg ha^−1^) ^a^	Manufacturer
Triclopyr acid ^b^	Trycera	3.4	Helena Agri-Enterprises, LLC, Collierville, TN, USA
Triclopyr amine + 2,4-D amine	Aquasweep	1.0 + 2.6	Nufarm Americas Inc. Alsip, IL, USA
Triclopyr amine ^b^	Garlon 3A	3.4	Corteva Agriscience, Indianapolis, IN, USA
Glufosinate	Finale	1.1	BASF Corp., Research Triangle Park, NC, USA

^a^ Rates are given in kg acid equivalent ha^−1^ with the exception of glufosinate which is presented in kg active ingredient ha^−1^. ^b^ Herbicides were applied with the addition of a non-ionic surfactant (AirCover, Winfield Solutions, St. Paul, MN, USA) at 0.5% (*v*/*v*) based on manufacturer recommendations.

## Data Availability

Data are contained within the article.
